# Recent Advances in DNA Lesion Mapping and Repair Mechanisms

**DOI:** 10.1021/acs.chemrestox.2c00307

**Published:** 2022-12-29

**Authors:** Arnold S. Groehler, Andrew H. Kellum

**Affiliations:** † Center for Genomic Integrity, Institute for Basic Science, Ulsan 44919 Republic of Korea; ‡ Department of Chemistry, University of California, Riverside, Riverside, California 92521-0403, United States

**Keywords:** DNA lesion, DNA repair, DNA quantification

## Abstract

Recognition and repair of DNA lesions are critical for cell survival. Herein, we highlight recent advances in the sequencing, repair mechanisms, and biological consequences of DNA lesions presented at the 2022 Fall American Chemical Society meeting.

Cellular DNA is under constant threat of chemical modifications by endogenous and exogenous sources. DNA lesions can interfere with the faithful expression and replication of genetic material, potentially leading to mutations, inhibition of cell division, and development of cancer. To avoid these detrimental consequences, cells have evolved dedicated DNA repair pathways to recognize and remove damaged DNA. New advances in mapping specific DNA lesions and discovering new DNA repair mechanisms were presented at the 2022 American Chemical Society meeting during the Chemical Toxicology Division thematic session “Chemical Biology on DNA Damage & Repair”. This symposium was organized by Yinsheng Wang from the University of California, Riverside and sponsored by Thermo Fisher Scientific Inc.

The introductory talk given by Kent Gates, Professor of Biochemistry and Chemistry at the University of Missouri, focused on the formation and repair of endogenous interstrand cross-links (ICLs) generated by abasic (AP) sites. Gates demonstrated that natural nucleobases adenine, cytosine, and guanine, as well as noncanonical nucleobases 2-aminopurine and *N*^4^-aminocytosine, can react with the ring-opened form of AP sites to form ICLs. Then the presentation focused on ICLs generated from AP sites and adenine, or dA-AP ICL. The dA-AP ICL was remarkably stable, formed in high yield and in a wide range of sequence contexts under physiological conditions. The presence of a site-specific dA-AP ICL within duplex DNA caused little structural perturbation to DNA double helix, as revealed by solution-phase NMR data. Gates then described how the DNA glycosylase NEIL3 unhooks dA-AP ICL in an *in vitro* model of a stalled replication fork, thus challenging the commonly perceived notion that glycosylases need to flip bases into their active site for repair.^1^ This repair pathway eliminated the need for endonucleolytic incisions and the associated repair of double-strand breaks (DSBs) during ICL repair.

The second talk presented by Linlin Zhao, Assistant Professor of Chemistry at the University of California Riverside, focused on exploring the chemistry of AP sites in mitochondrial DNA (mtDNA). Mitochondria lack some repair pathways that are present in the nucleus (e.g., nucleotide excision repair) and degrade damaged DNA by mitochondrial DNA turnover processes. However, the mechanism of mtDNA degradation is not fully elucidated. Zhao hypothesized that mitochondrial transcription factor A (TFAM) promoted the degradation of AP sites in mtDNA due to the binding of a large number of TFAM molecules per mtDNA, as well as the high percentage of lysine residues that could facilitate DNA strand cleavage at AP sites via Schiff base chemistry. Zhao demonstrated that TFAM forms DNA protein cross-links (DPCs) with AP sites in mtDNA. In the absence of reducing agents, the generated DPC promoted strand cleavage at AP sites within mtDNA and reduced the half-life of mtDNA with AP sites.^2^ Zhao then presented a small-molecule labeling approach for enriching and mapping AP sites in mtDNA. This sequencing approach revealed the presence of AP sites within low-TFAM coverage G-quadruplex (G4) regions of mtDNA. Together, the research showed a novel role of TFAM in facilitating the cleavage of damaged mtDNA containing AP sites.

Eukaryotic genomic DNA is packaged into chromatin, with the DNA wrapped ∼1.7 times around an octameric histone core forming nucleosome core particles (NCPs). Sarah Delaney, Professor of Chemistry at Brown University, discussed the effects histone protein variants have on the repair of DNA lesions in NCPs by base excision repair (BER). The NCPs were assembled using the Widom 601 DNA sequence containing DNA lesions such as uracil and 1,*N*^6^-ethenoadenine (εA) with recombinant histone proteins. For example, NCPs composed of macroH2A histone variants formed a mixture of octasomes and hexasomes, the latter facilitating the efficient removal of uracil by uracil-DNA glycosylase (UDG) and single-strand-selective monofunctional uracil-DNA glycosylase (SMUG1) in intermediate and low solvent-accessible regions and the dyad axis. Globular H2B histones missing the N-terminal tail enhanced excision of εA lesions by DNA-3-methyladenine glycosylase (AAG) due to unwrapping of the DNA in the DNA entry/exit region of the NCP, while globular H3 histones inhibited εA excision by altering DNA periodicity.^3^ Taken together, the repair of damaged DNA on NCPs is influenced by the structural and dynamic constraints of DNA–histone interactions and any factor that causes disruption to these interactions.

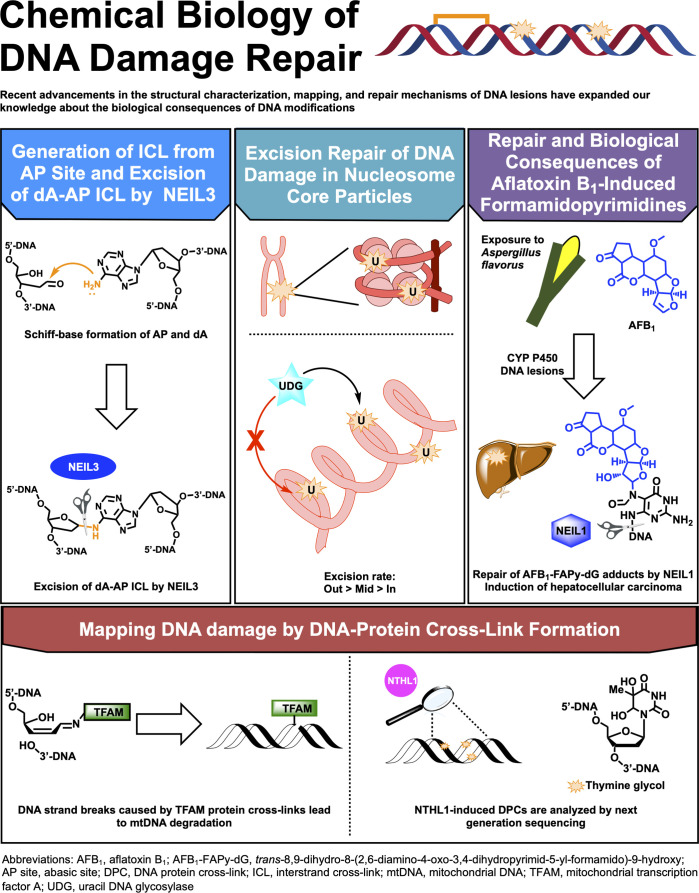



Prof. R. Stephen Lloyd of the Oregon Institute of Occupational Health Sciences presented on the relationship between aflatoxin B1 (AFB_1_) exposure and the induction of hepatocellular carcinoma (HCC). AFB_1_ alkylates the *N*7 position of dG, followed by a nonenzymatic ring opening to yield a formamidopyrimidine (AFB_1_–FAPy-dG) adduct. *In vitro* polymerase bypass assays revealed that AFB_1_–FAPy-dG completely blocks replicative Pol δ and the translesion synthesis (TLS) Pol κ, η, and λ but is bypassed by TLS Pol ζ in an error-prone manner. AFB_1_–FAPy-dG is excised by the BER enzyme endonuclease VIII-like 1 (NEIL1). *NEIL1*^–/–^ mice exposed to AFB_1_ accumulated 3-fold more AFB_1_–FAPy-dG lesions and had a 3.4-fold increased risk of developing HCC compared to wild-type mice. Furthermore, *NEIL1*^*–/–*^ mice exposed to AFB_1_ had a 17-fold increase in single-base mutations. Characterization of NEIL1 polymorphic variants that are found in East Asia revealed decreased activity in the P68H and that the A51V was temperature sensitive, suggesting it may also be a pathologic variant.^4^ Although these variants occur at a low frequency in the general East Asian population, all three NEIL1 polymorphic variants were identified in a small cohort of early onset HCC patients from the Qidong province in China, thus potentially linking NEIL1 variants with increased risk of liver cancer.

The final talk of the symposium given by Yinsheng Wang, Professor of Chemistry at the University of California, Riverside, focused on genome-wide mapping of thymine glycol using a novel DNA–protein cross-linking sequencing (DPC-Seq) method.^5^ This method utilizes the bifunctional DNA glycosylase NTHL1 to locate and excise thymine glycol, followed by reduction of the Schiff base intermediate in the DPC formed with the generated AP site to yield a stable complex capable of enrichment for sequencing. Their results revealed that thymine glycol was depleted in regions of active transcription but enriched at nucleosome-binding sites as well as intron and intergenic regions. Furthermore, Wang demonstrated that digested DPCs resulting from thymine glycol and NTHL1 inhibited the Q5 polymerase, potentially allowing for single-nucleotide resolution mapping of the lesion in genomic DNA.

Cellular DNA is under constant threat of chemical modification, requiring dedicated DNA repair pathways to restore DNA to its original state. Developing new methodologies to quantify and accurately map DNA lesions is crucial to understanding the toxicity of endogenous and exogenous DNA damaging agents. Furthermore, elucidating the repair mechanism(s) of these DNA lesions will allow for a better understanding of the etiology of diseases such as cancer.
